# Differential microbial composition in parasitic vs. questing ticks based on 16S next-generation sequencing

**DOI:** 10.3389/fmicb.2023.1264939

**Published:** 2023-12-07

**Authors:** Lin Zhang, Jing Han, Qi Zhou, Zhen He, Shi-Wei Sun, Rui Li, Rui-Shan Li, Wen-Kai Zhang, Yu-Hua Wang, Lin-Li Xu, Zhen-Hua Lu, Zhong-Jun Shao

**Affiliations:** Department of Epidemiology, Ministry of Education Key Lab of Hazard Assessment and Control in Special Operational Environment, School of Public Health, Air Force Medical University, Xi’an, Shaanxi, China

**Keywords:** parasitic tick, questing tick, symbiotic bacteria, microbiome analysis, network diagram

## Abstract

**Introduction:**

As tick-borne diseases rise to become the second most prevalent arthropod-transmitted disease globally, the increasing investigations focus on ticks correspondingly. Factors contributed to this increase include anthropogenic influences, changes in vertebrate faunal composition, social-recreational shifts, and climatic variation. Employing the 16S gene sequence method in next-generation sequencing (NGS) allows comprehensive pathogen identification in samples, facilitating the development of refined approaches to tick research omnidirectionally.

**Methods:**

In our survey, we compared the microbial richness and biological diversity of ticks in Wuwei City, Gansu province, differentiating between questing ticks found in grass and parasitic ticks collected from sheep based on 16S NGS method.

**Results:**

The results show *Rickettsia*, *Coxiella*, and *Francisella* were detected in all 50 *Dermacentor nuttalli* samples, suggesting that the co-infection may be linked to specific symbiotic bacteria in ticks. Our findings reveal significant differences in the composition and diversity of microorganisms, with the *Friedmanniella* and *Bordetella* genera existing more prevalent in parasitic ticks than in questing ticks (*p* < 0.05). Additionally, the network analysis demonstrates that the interactions among bacterial genera can be either promotive or inhibitive in ticks exhibiting different lifestyles with the correlation index |*r*| > 0.6. For instance, *Francisella* restrains the development of 10 other bacteria in parasitic ticks, whereas *Phyllobacterium* and *Arthrobacter* enhance colonization across all tick species.

**Discussion:**

By leveraging NGS techniques, our study reveals a high degree of species and phylogenetic diversity within the tick microbiome. It further highlights the potential to investigate the interplay between bacterial genera in both parasitic and questing ticks residing in identical habitat environments.

## 1 Introduction

Tick-borne diseases remain a longstanding and burgeoning global health concern, with disease incidences seeing a dramatic increase since the 20th century (Rodino et al., 2020). This trend is evident worldwide and reflected in the rising number of tick-borne encephalitis and hemorrhagic fever cases reported across Europe and Asia (Rochlin and Toledo, 2020). In China, for instance, tick-borne diseases attributed to over 40 diverse pathogens have proliferated across several provinces, including Anhui, Henan, Inner Mongolia, Tibet, Zhejiang, and Liaoning, since initial reporting in 1982 with the amplification of epidemic area and infected human amount (Lan-Hua and Yi, 2019). Notwithstanding these broad findings, studies specific to Gansu Province remain scarce. However, the importance of investigating the intricate role of microbiology in environmental contexts, given ticks’ critical function as bacterial vectors, is well-documented in other regions of China.

A deeper understanding of the composition and diversity of microbiomes influencing pathogen transmission necessitates a nuanced exploration of both parasitic (feeding) ticks and questing (off-host) ticks. Differentiating these two tick lifestyles may yield crucial insights into disease manifestation in relation to varying host or off-host circumstances (Ciebiera et al., 2021), although the interplay between tick communities and the pathogens they carry remains somewhat nebulous (Kahl, 2018). Although parasitic ticks predominantly inhabit the body surfaces of hosts such as humans and domestic animals, facilitating pathogen transmission, the comparative studies of these ticks with their counterparts living in wild grass and scrub are hampered by unilateral approaches that focus narrowly on specific tick categories and pathogen transmission routes.

In the last 20 years, various techniques have been employed to elucidate the microbial composition within tick communities. The advent of next-generation sequencing (NGS) has accelerated pathogen identification, providing a rapid and efficient means to comprehensively explore pathogen community structure (Sanschagrin and Yergeau, 2014). Utilized extensively in bacterial gene detection, NGS offers high efficiency and accuracy in identifying microbes from large sample sets in a short time frame (Chaorattanakawee et al., 2022). The conservation and variability within the small subunit ribosomal RNA gene, especially the 16S rRNA gene, permit the design of unique primers for specific bacterial species, making NGS a powerful tool for disease surveillance and comprehensive microbial taxonomic identification (Rodino et al., 2021).

For this study, we selected Wuwei City in Gansu province, China, known for its rich species diversity, as our sampling site (Sun et al., 2016). Previous reports have identified new species like *Ornithodoros huajianensis* in the city’s Mongolian marmots (Sun et al., 2019), as well as the presence of *Babesia* spp. and *Theileria* spp. in cattle (Sun et al., 2020). Our research aims to elucidate the differences in microbiomes and tick-borne pathogens in Wuwei City, Gansu Province. A thorough investigation of both questing and parasitic ticks will be conducted, intending to bridge the gap in current studies that overlook the microbiome composition and bacterial transmission differences between these two lifestyle ticks in natural environments. Ultimately, we intend to enhance understanding of tick-borne pathogens by detailing every procedure of this study, from tick collection to NGS analysis, with a focus on characterizing the questing ticks and the feeding ticks from sheep in Wuwei City.

## 2 Materials and methods

### 2.1 Ticks collection and morphological observation

All ticks were collected from Wuwei City (102.65°E, 37.94°N), Gansu province, in May 2022. Feeding ticks were carefully extracted from sheep using tweezers while questing ticks were gathered from grassland using the Flag Cloth Law method, which involves dragging a 90 cm × 60 cm white flannelette flag across the surface. It should be noted that the sampling of both questing and feeding ticks was opportunistic and was therefore not standardized with respect to collection area or time.

Following collection, all ticks were immediately stored in tubes with perforated lids and refrigerated at 4°C to minimize tick mortality. Afterward, ticks’ surfaces were cleaned using 75% ethyl alcohol, and identification was carried out with a stereoscopic microscope (RH-2000, HIROX, Japan), based on observed structural characteristics of the ticks.

### 2.2 Nucleic acid extraction

Three ticks, morphologically identified as *Dermacentor* at the *genus* level, were placed in a tube and labeled according to the collecting date and lifestyles for subsequent operations. Each tube was injected with 1 mL of Stroke-Physiological Saline Solution (SPSS, 0.9% NaCl), and 3–4 0.2 mm steel balls were added before the samples were ground at a frequency of 65 Hz for 500 s. Following this, the samples were incubated in a dry water bath for 10 min at a consistent temperature of 55°C, and the supernatant fluid was carefully transferred to a new tube after a rapid centrifugation at 12,000 rpm for 1 min in a standard centrifuge. Nucleic acid extraction was then performed using the Ex-DNA/RNA nucleic acid extraction kit (TIAN LONG, China) according to the manufacturer’s instructions (Shen et al., 2020), and the extracted samples were stored at −20°C for further analysis.

### 2.3 PCR amplification and identification

Tick identification was achieved by amplifying DNA using PCR, specifically targeting the mitochondrial gene cytochrome c oxidase I (COI; Lv et al., 2014). The primers used for the molecular biological identification of ticks were COI (Forward primer: 5′-GGAACAATATATTTAATTTTTGG-3′, Reverse primer: 5′-ATCTATCCCTACTGTAAATATATG-3′).

The PCR reactions contained 40 ng of genomic DNA and were conducted in 50 μL reaction volumes including 25 μL 2× DreamTaq PCR Master Mix (Thermo, America), 2 μL of each primer (10 μmol/L), 2 μL of DNA (20 ng/μL), and 19 μL of ddH_2_O. Reactions were performed in a thermocycler under the following conditions: an initial preheating step at 95°C for 3 min, followed by 35 cycles of denaturation at 95°C for 30 s; annealing at 45°C for 30 s; extension at 72°C for 1 min; and a final extension at 72°C for 10 min.

The PCR products were subjected to 1.2% agarose gel electrophoresis (AGE) under specific conditions, including a voltage of 220 V, a current of 400 mA, and a duration of 30 min. Following this, the gel was imaged using a gel imager to visually represent the DNA fragments. For precise identification of the tick species, the nucleotide sequence of the PCR products was determined using Sanger sequencing, which was facilitated by AuGCT DNA-SYN Biotechnology Co., Ltd. located in Beijing, China.

### 2.4 Library preparation and sequencing

Aimed to amplify the sequences of species-specific for the 16S rRNA gene of the ribosome’s large subunit, the methods followed involved the generation of a DNA library using QIAseq FX DNA Library Kit (QIAGEN, Germany), as per the manufacturer’s instructions, before diluting the nucleic acid for subsequent sequencing, so that the concentration could be detected by the Qubit-4 (Thermo, America).

The primary protocol encompassed several steps, including library preparation via the fusion method, amplicon purification, quantification, and pooling. The final products were individually validated using AGE and any samples with concentrations falling below the standard 50 ng/μL, as defined by the Nextera XT Index Kit v2 (Illumina), were deemed unqualified and hence excluded from the pool. All pooled DNA samples were then subjected to paired-end sequencing, utilizing the MiSeq Reagent Kit V3 on the Illumina HiSeq platform (Odendaal et al., 2022). This sequencing process adhered strictly to the manufacturer’s instructions and was characterized by an insert size of 350 bp and a read length of 250 bp.

### 2.5 Sequence data analysis

The barcode-based data were analyzed using the Quantitative Insights Into Microbial Ecology version2 (QIIME2) software suite (Caporaso et al., 2010), which provides a comprehensive software environment, data standards, and tool wrappers. The sequence data, presented in FASTQ format, were demultiplexed and consolidated into the same directory before input as QIIME artifacts (.qza) or QIIME visualizations (.qzv). After importing the paired-end reads from the original DNA fragments into the virtual machine, we utilized the DADA2 method to denoise data by truncating both forward and reverse sequences at 230 base pairs each. Following the DADA2 protocol, the sequences were sorted and grouped into Autosave (ASV), which is replaced by Operational Taxonomic Unit (OTU) in the following. The sequences were matched against the Silva database according to the Uchime algorithm (Edgar et al., 2011). Upon creation or acquisition of the classifier artifact, the “*qiime taxa barplot”* command was used to perform taxonomic analysis of the OTU representative sequences. The bacterial composition of each sample was visualized in a bar figure using the R packages ggplot2 and RColorBrewer (Wickham, 2009; Neuwirth, 2022).

### 2.6 The alpha and beta diversity analysis

For single-sample diversity analysis or alpha diversity, we determined sufficient sequencing depth based on rarefaction curves for the observed number of OTUs across all samples shown as illustrated in the Good’s coverage. After the sequences of all samples were randomly drawn to a uniform data volume, we calculated the number of unique OTUs, the community richness as determined by the Shannon estimator, and the community evenness as indicated by the Pielou index in each sample using the R package Vegan (Dixon, 2003). The Wilcoxon rank-sum test was employed to assess whether these indices differed significantly and the analysis of similarities (ANOSIM) was used to quantify the variation in bacterial composition resulting from different tick lifestyles using package Vegan.

Following the exclusion of *Rickettsiaceae*, the abundance of bacteria in samples was recalculated and beta diversity analyses were performed at the *family* level using the R packages Vegan, phyloseq, and ggplot2 (McMurdie and Holmes, 2013). Weighted and unweighted UniFrac analyses were utilized to examine diversity and to compare different groups, which were subsequently plotted in a principal coordinate analysis (PCoA). The Welch’s *t*-test was applied to assess differences in bacterial composition between two tick lifestyles. Variations in bacterial composition between groups were visualized using STAMP software (Parks et al., 2014). Lastly, to identify microbial taxa with significant differences between Groups A and B from *phylum* to *species* level, we employed Linear Discriminant Analysis (LDA) Effect Size (LEfSe; Segata et al., 2011). A threshold LDA score of >2 was used within 95% confidence intervals.^1^

### 2.7 Phylogenetic analysis and network relationship

The representative COI gene sequences and pathogen reads obtained were cross-referenced with existing results in GenBank, employing the Basic Local Alignment Search Tool (BLAST) search engine provided by the National Center for Biotechnology Information (NCBI).^2^ Multiple sequence alignment was conducted using the ClustalW algorithm within the MEGA-11 software suite, applying default parameters. The phylogenetic tree was then constructed using the Kimura two-parameter model of Neighbor-Joining method based on MEGA-11, with bootstrap values estimated from 1,000 replicates.

The Spearman rank correlation test was conducted to present the interrelationship between two bacteria in samples using the R packages Hmisc and Igraph (Csardi and Nepusz, 2005), and the networks were constructed to visualize relationships between OTUs at the *genus* level. The two bacteria were linked by red or blue lines which represented a positive or negative correlativity, respectively, a process facilitated by Gephi software.^3^ OTUs that were either unassigned or occurred only once were disregarded at the *genus* level and a cut-off value was established for reads to eliminate pathogens. Microbial occurrences that represented less than 10% of the frequency distribution of reads in a microorganism were discarded, and the correlation coefficient (|*r*|-value) was set at >0.6 with a 95% confidence level (*p* < 0.05).

## 3 Results

### 3.1 Tick identification

**Figure 1:**
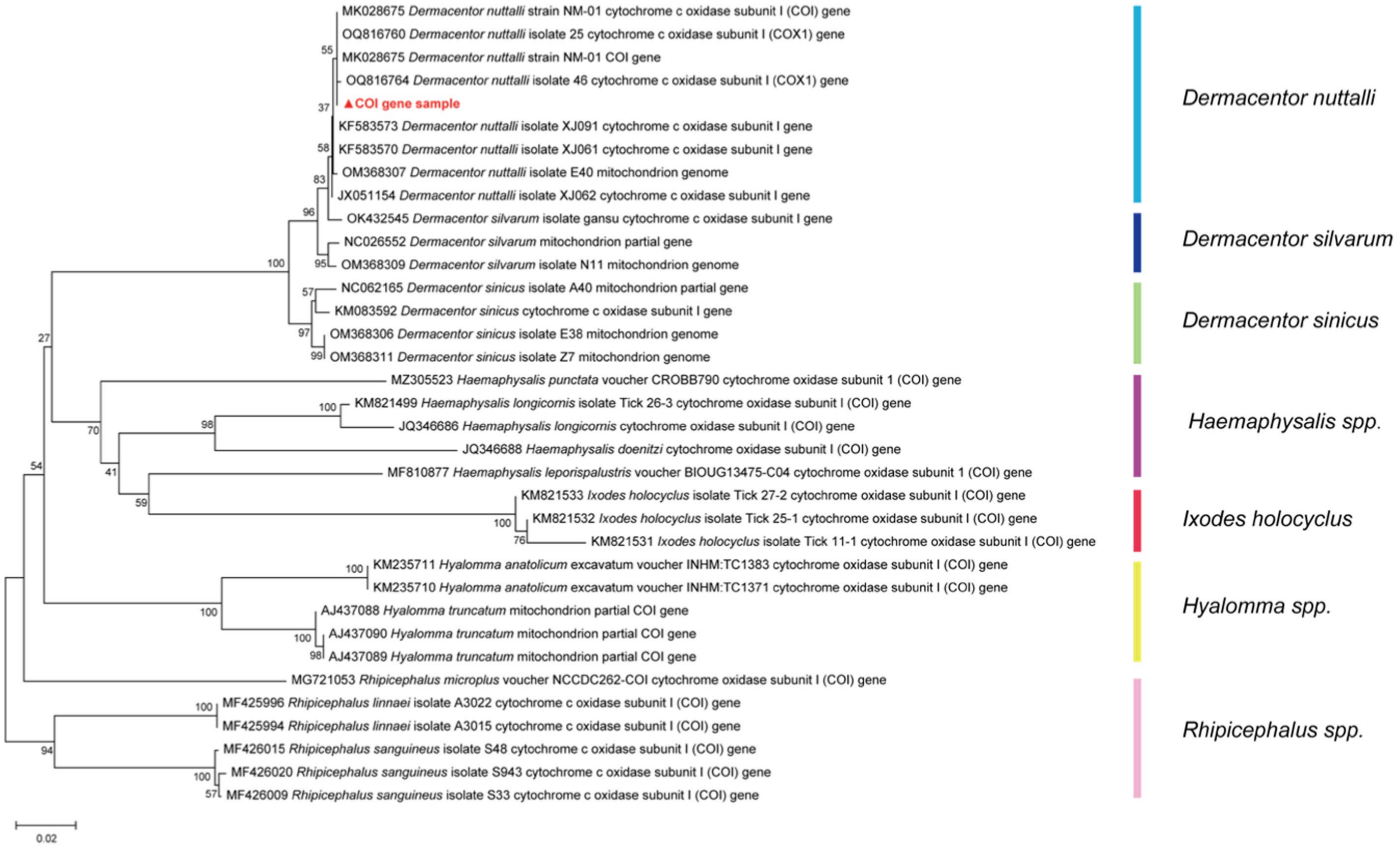
The phylogenetic tree of the COI gene with NCBI Blast results of tick species, was constructed based on the Kimura two-parameter model of Neighbor-Joining method using 1,000 repeated estimated bootstrap values.

The ticks were categorized into two groups based on their mode of capture: Group A consisted of feeding ticks, while Group B comprised questing ticks. A total of 150 adult ticks were identified as *Dermacentor nuttalli* (Acari: *Ixodidae*) according to statistics, confirmed through morphological identifications and a PCR assay of the species-specific COI region, which a high identity range of 92.66–99.75%. The phylogeny is shown in Figure 1.

### 3.2 Pathogen detection

Prior to the 16S metagenomics sequencing, our laboratory conducted nested PCR assays using specific primers, through which five pathogens, namely *Rickettsia*, *Anaplasma*, *Borrelia burgdorferi*, *Babesia*, and *Bartonella*, were detected. The primer sequences are outlined in Table 1, displayed from 5′ to 3′ for both forward and reverse sequences.

**Table 1:** The primer sequence of five pathogens from 5′ to 3′.

Target genes and primer sequences used for nested PCR				
Pathogen	Target gene	Primer name	Sequence	Tm (T/°C)
*Rickettsia*	ompA	190.70f	5′-ATGGCGAATATTTCTCCAAAA-3′	54
		190.602r	5′-AGTGCAGCATTCGCTCCCCCT-3′	
		38 s1	5′-AAAACCGCTTTATTCACC-3	
		384r1	5′-GGCAACAAGTTACCTCCT-3′	
*Anaplasma*	16S rRNA	Eh-out1	5′-TTGAGAGTTTGATCCTGGCTCAGAACG-3′	54
		Eh-out2	5′-CACCTCTACACTAGGAATTCCGCTATC-3″	
		Eh-gs1	5′-GTAATAACTGTATAATCCCTG-3	
		Eh-gs2	5′-GTACCGTCATTATCTTCCCTA-3″	
*Borrelia burgdorferi*	ITS	23 s3	5′-CGACCTTCTTCGCCTTAAAGC-3	59
		23Sa	5′-TAAGCTGACTAATACTAATTACCC-3′	
		23 s5	5′-CTGCGAGTTCGCGGGAGA-3′	
		23S6	5′-TCCTAGGCATTCACCATA-3	
*Babesia*	18S rRNA	Bab1	5′-CTTAGTATAAGCTTTTATACAGC-3″	54
		Bab4	5′-ATAGGTCAGAAACTTGAATGATACA-3′	
		Bab2	5′-GTTATAGTTTATTTGATGTTCGTTT-3″	
		Bab3	5′-AAGCCATGCGATTCGCTAAT-3′	
*Bartonella*	ITS	302F	5′-YCTTCGTTTCTCTTTCTTCA-3′	55
		473R	5′-AACCAACTGAGCTACAAGCC-3′	
		311F	5′-CTCTTTCTTCAGATGATGATCC-3′	
		448R	5′-GGATAAACCGGAAAACCTTC-3′	

Of all the tested nucleic acid samples, the prevalence rates for *Rickettsia* and *Anaplasma* stood at 32.67 and 4.67%, respectively, surpassing those of the other three pathogens. Notably, *Borrelia burgdorferi*, *Babesia*, and *Bartonella* were not detected using conventional PCR assays. A subset of the samples (4.67%) demonstrated detected with *Rickettsia* and *Anaplasma* simultaneously. Results of the positive rate are presented in Supplementary Table 1.

### 3.3 Data general statistic

**Figure 2:**
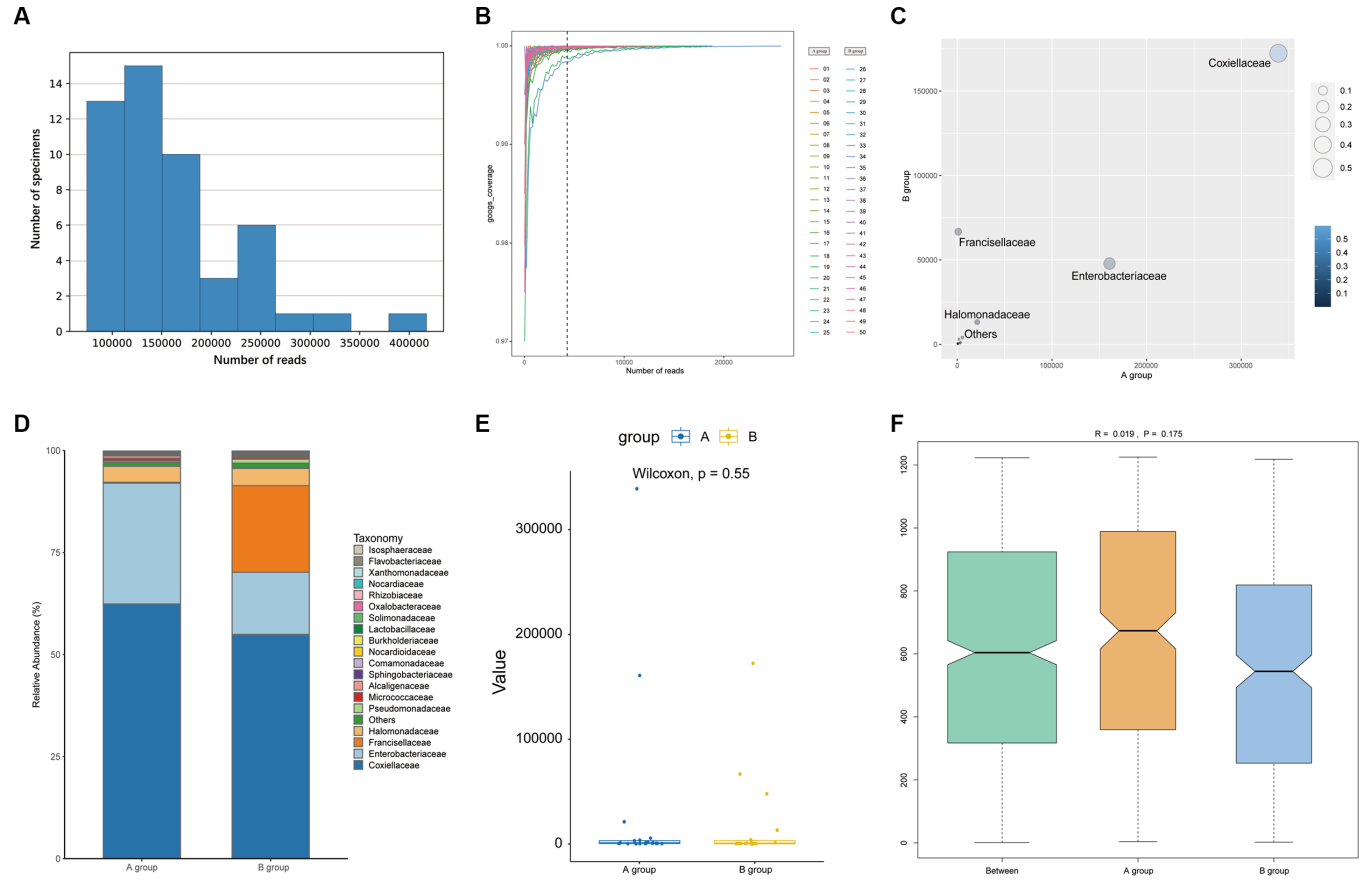
The plots show the information of reads **(A,B)** and the comparison between groups after removing *Rickettsiaceae* at the *family* level **(C–F)**. **(A)** The bar shows the reads in samples. **(B)** Rarefaction curves estimated from reads obtained. **(C)** The number of reads of each *family* of bacteria is plotted. **(D)** The bar plot shows the microbial community between groups. **(E)** A Wilcoxon test of OTUs between two groups with a box plot. **(F)** The ANOSIM analysis between groups (pseudo-*R* = 0.019, *p* = 0.175).

Using the Illumina HiSeq platform and after eliminating low-quality sequences, 5,480,349 reads were obtained in the data analysis. The bacterial genera were determined by comparing the taxonomic profiles at the *genus* level against the Silva database. Approximately 8 × 10^6^ paired-end V3-V4 16S reads were procured from all samples, with the read count varying from 7,332 to 417,497 and Supplementary Table 2 included 16S NGS sample productions. After pre-processed (merged, quality filtered, and removal of singletons and chimeras) and post-processed, a cumulative total of 5 million sequences were obtained for the samples and their replicates. The average number of sequences was 109,607 and a range from 42,877 to 257,450 as illustrated in Figure 2A. The rarefaction curves, which level off well before 10,000 reads (Figure 2B), indicate that the number of reads is sufficient to compile a reliable list of bacterial genera. The raw data had uploaded to NCBI with the Bio-Project Accession Number PRJNA1015185.

### 3.4 Taxonomy and group comparison

**Figure 3:**
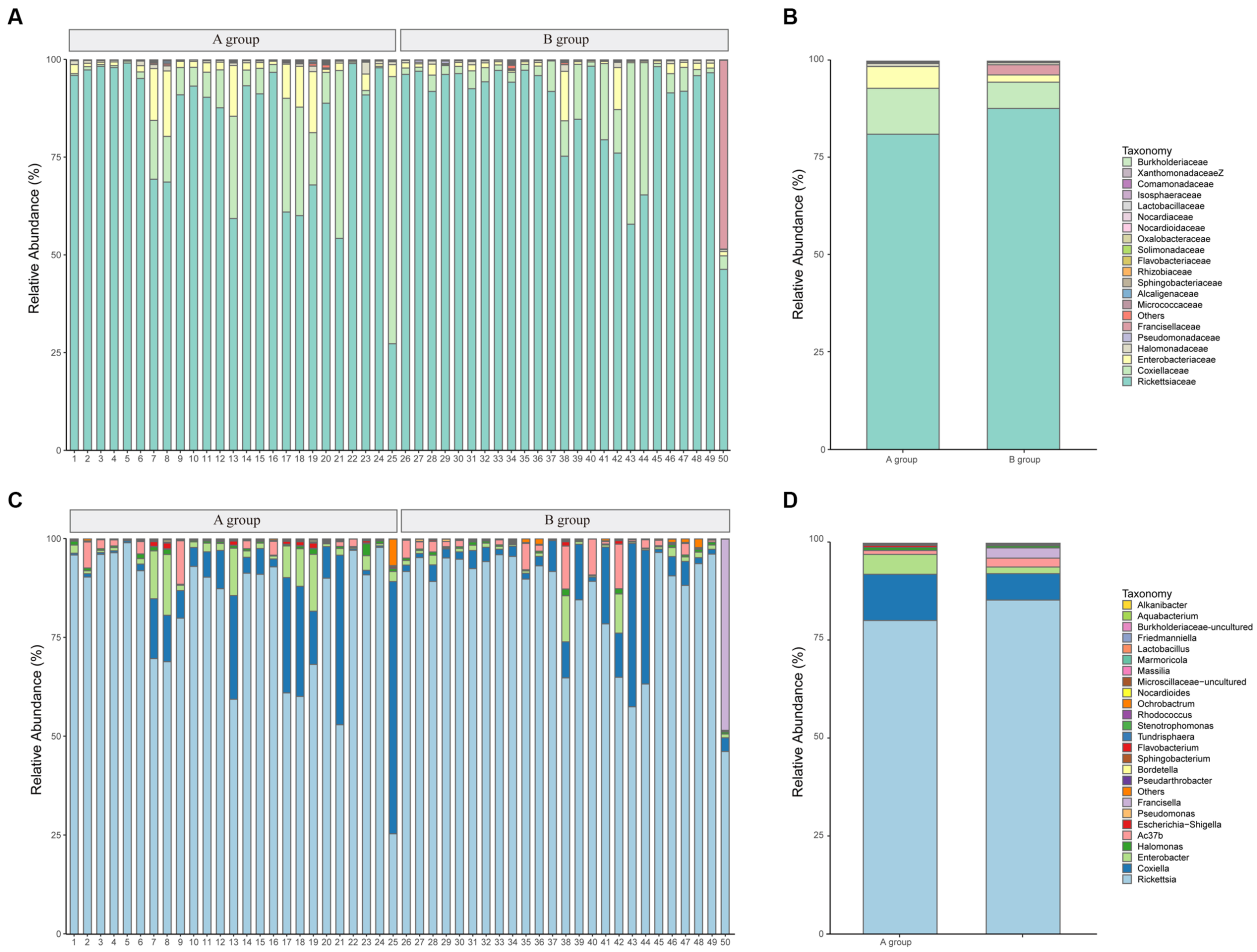
The microbial community bar plots of bacteria comparison. **(A)** The top 20 communities at the *family* level for samples. **(B)** The top 20 communities at the *family* level with groups. **(C)** The top 25 communities at the *genus* level for samples. **(D)** The top 25 communities at the *genus* level with groups.

At the *family* level, *Rickettsiaceae*, *Coxiellaceae*, and *Enterobacteriaceae* represented an average of 84.21, 9.42, and 3.84%, respectively. The top 20 taxonomics for each sample are illustrated in Figure 3A. Although *Rickettsiaceae* is predominant in most samples, the *Coxiellaceae* and the *Fancisellaceae* are significantly prevalent in samples number 25 (33,450 reads, 68.18%) and 50 (65,231 reads, 48.29%) respectively. As shown in Figure 3B, *Rickettsiaceae* comprises 2,330,700 (81.11%) and 2,241,292 (87.71%) of the reads in Groups A and B, respectively. Notably, *Coxiellaceae* and *Enterobacteriaceae* in Group A (339,077 reads, 11.80%; 160,804 reads, 5.60%) have approximately double and triple the reads in Group B (172,405 reads, 6.75%, 47,821 reads, 1.87%) respectively. In contrast, the *Francisellaceae family*, although less prominent, shows a slight distinction between the groups, with Group B (66,723 reads, 2.61%) having more reads and proportions than Group A (1,187 reads, 0.04%).

And at the *genus* level, *Rickettsia* is the dominant *genus* across all samples, comprising between 25.36 and 98.95% of the total community (average: 82.63%), followed by *Coxiella* and *Enterobacter*, which account for averages of 9.42, and 3.49%, respectively. Figure 3C provides a visual representation of the relative abundance of the top 25 species at the *genus* level. The figure illustrates that the *genus*-level classifications of *Rickettsia* and *Ac37b* collectively constitute the *family*-level classification of *Rickettsiaceae*. The comparison of the top 25 species between groups is shown in Figure 3D.

Regarding alpha diversity, there were no significant differences between groups for either Shannon Index (*W* = 359, *p* = 0.375) or Simpson Index (*W* = 353, *p* = 0.441), despite the range of the two Index being wider for Group A compared to Group B, as presented in Supplementary Figure 1. The Wilcoxon rank test of other indexes also had no statistical differences which are shown in Supplementary Table 3. There were no significant differences in bacterial microbiome compositions between parasitic and questing ticks according to ANOSIM (pseudo-*R* = 0.019, *p* = 0.175), as illustrated previously in Figure 2F.

### 3.5 The diversity removed *Rickettsiaceae*

**Figure 4:**
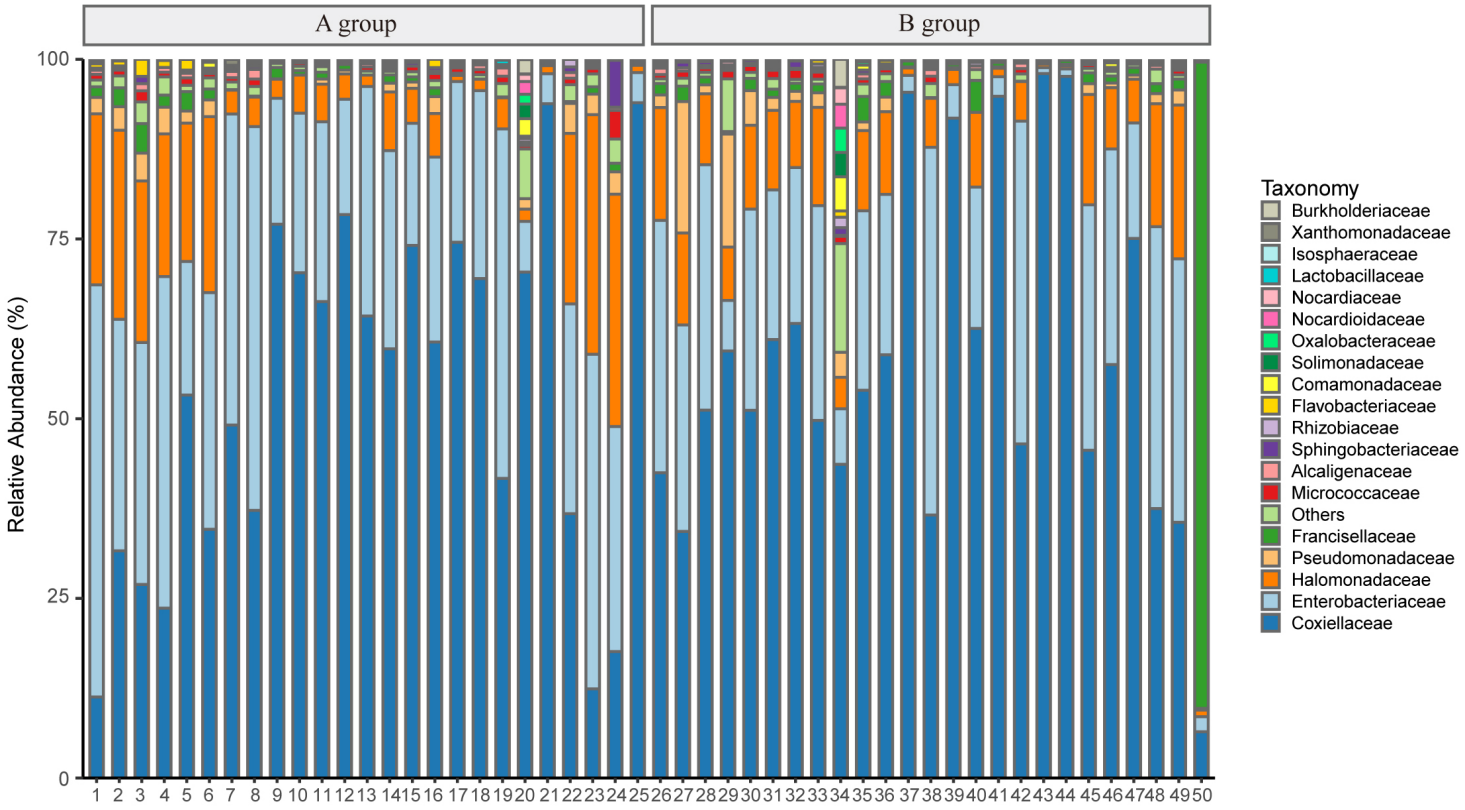
The plot shows the relative abundance of taxonomy after removing *Rickettsiaceae* at the *family* level.

However, after removing *Rickettsiaceae* computationally, the composition unveils noteworthy variations between groups (Figure 2C). It is apparent that Figure 2D explicitly presents the dominant role of *Coxiellaceae* within two groups (A: 339,077 reads, 62.46%; B: 172,405 reads, 54.88%), and subsequently, a significant disparity in the presence of *Enterobacteriaceae* (A: 160,804 reads, 29.62%; B: 47,821 reads, 15.22%) is discernible in Figure 4. Interestingly, while the relative abundance of *Francisellaceae* in Group B (21.24%) surpasses that in Group A (0.22%), no statistical difference in microbiome composition at the *family* level is noted (*p =* 0.55, Wilcoxon rank-sum test) as depicted in Figure 2E. A comparative analysis of the *Ixodes* microbiome diversity reveals greater species evenness in Group B (0.628) relative to Group A (0.520) based on the Simpson index, while the Shannon index suggests that the species richness in Group A (1.044) is lower than in Group B (1.363) as shown in Supplementary Table 4.

**Figure 5:**
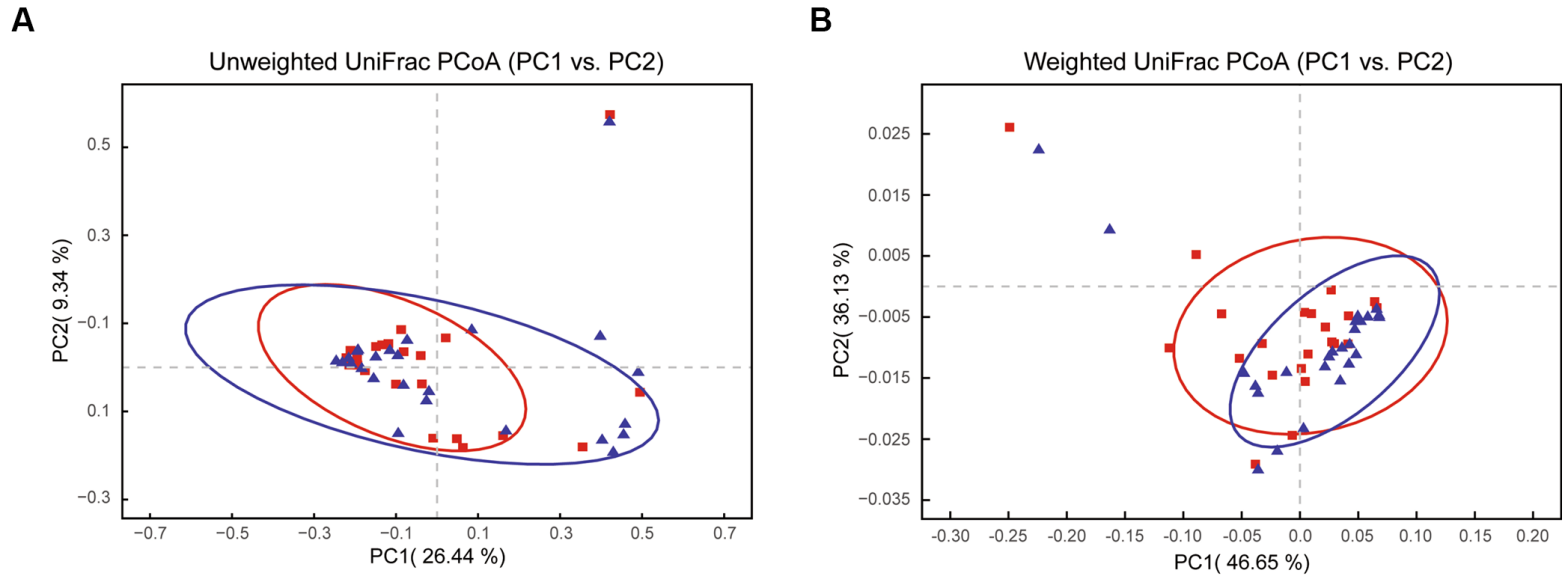
The plot shows PCoA results between groups after removing *Rickettsiaceae*. **(A)** The Unweighted UniFrac PCoA graph shows PC1 (26.44% variation) and PC2 (9.34% variation). **(B)** The Weighted UniFrac PCoA graph shows PC1 (46.65% variation) and PC2 (36.13% variation).

**Figure 6:**
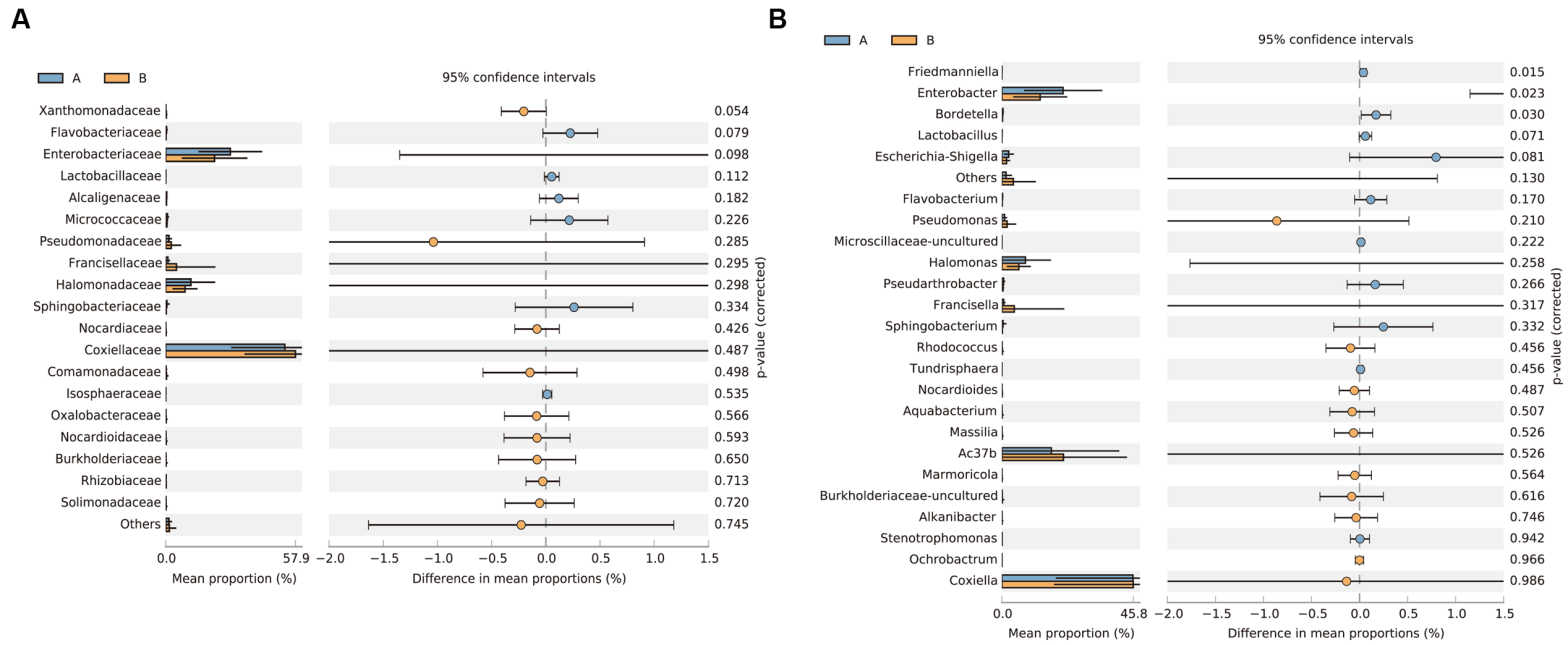
The plot shows the results of the Welch’s *t*-test between groups after removing *Rickettsiaceae*. **(A)** Differences of bacterial composition in 95% confidence intervals at the *family* level. **(B)** Differences of bacterial composition in 95% confidence intervals at the *genus* level.

**Figure 7:**
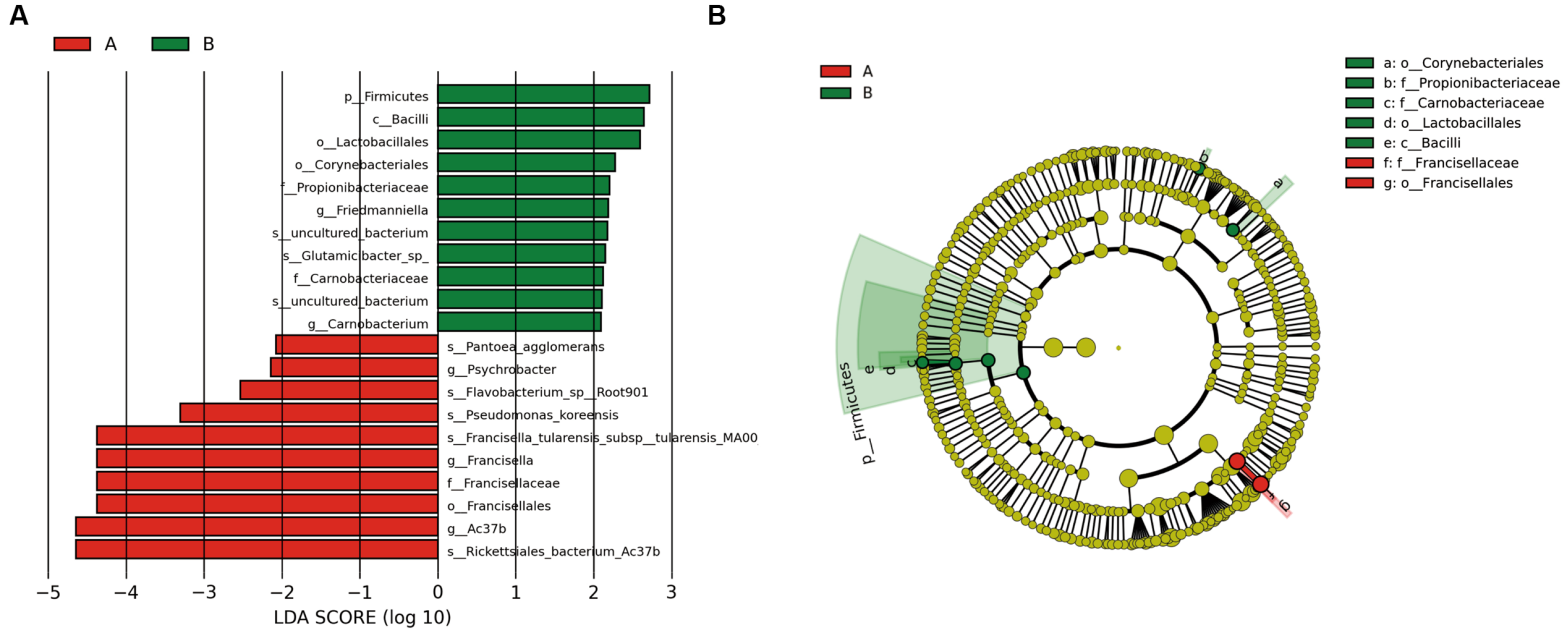
The plot shows LEFSe results between groups after removing *Rickettsiaceae*. **(A)** The bar plot explains the contribution degree of distinct species (LDA score > 2, *p* < 0.05). **(B)** The inner to outer circle of the cladistic map of distinct species evolution represents the classification level from *phylum* to *family*.

Principal coordinate analysis identifies the species basically similar between the groups. The Unweighted UniFrac PCoA further reveals that PC1 (26.44%) surpasses PC2 (9.34%), and in the Weighted UniFrac PCoA plot, PC2 (36.13%) is inferior to PC1 (46.65%), as represented in Figures 5A,B. A comparison of the relative abundance of the top 20 bacterial genera using the Welch’s *t*-test (two sides), identifies no significant differences at the *family* level within the 95% confidence intervals, as shown in Figure 6A. While *Enterobacteriaceae* is marginally more prevalent in Group A than Group B, the mean proportion of *Francisellaceae* and *Coxiellaceae* is comparatively diminished. However, substantial differences become apparent at the *genus* level as shown in Figure 6B, with the result of the relative abundance of *Friedmanniella* and *Bordetella* being elevated in parasitic ticks as compared to questing ticks. Figure 7A demonstrates differences among specimens at *p* < 0.05 as determined by LEfSe analysis, and a slight discrepancy is detectable within 95% confidence intervals from *phylum* to *species* in Figure 7B, with 11 taxa in Group B and 10 taxa in Group A detected at higher relative abundance.

### 3.6 Phylogenetic tree and bacterial network

**Figure 8:**
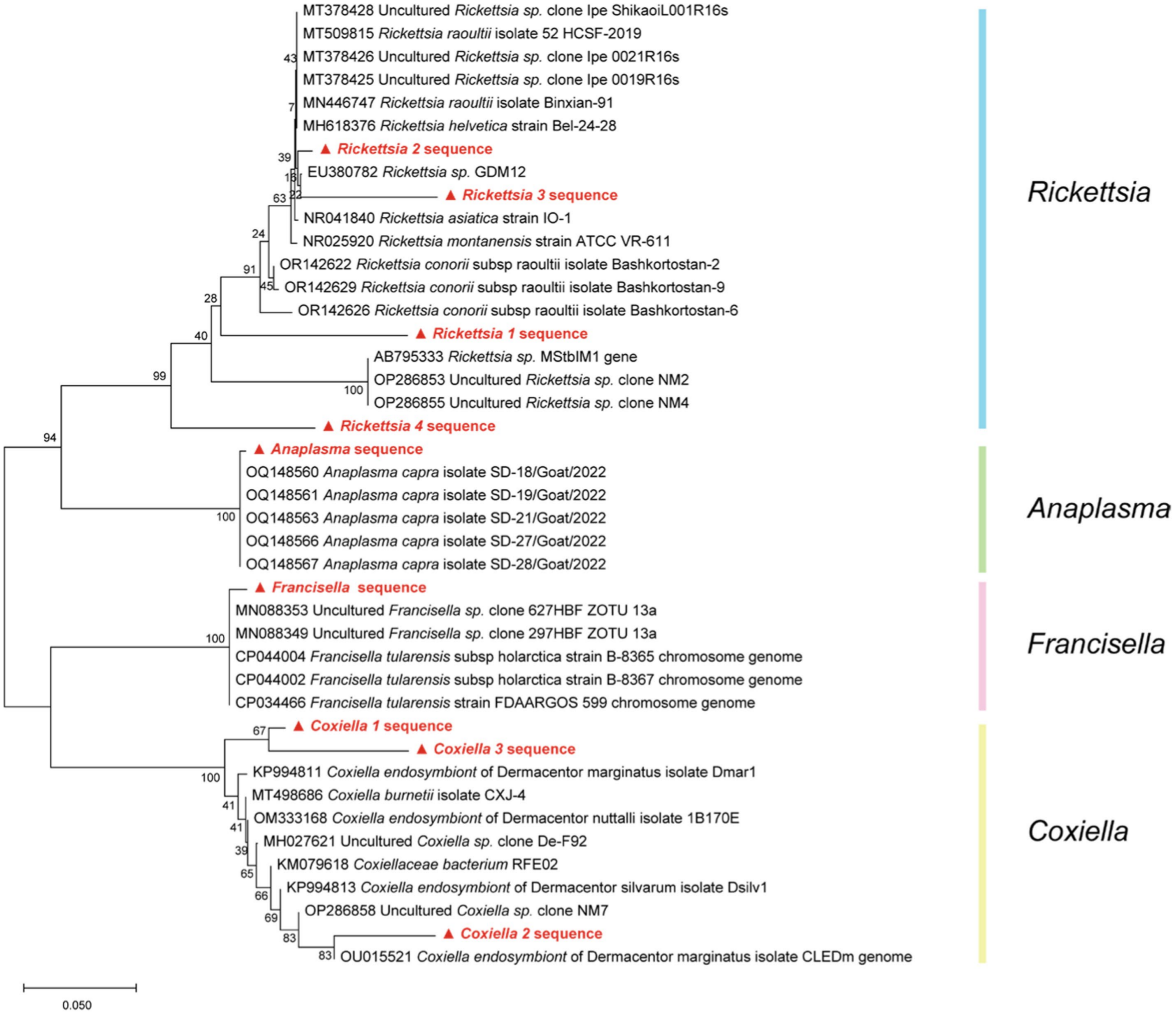
Based on the Kimura two-parameter model of Neighbor-Joining method, the phylogenetic tree of four pathogens (*Rickettsia*, *Anaplasma*, *Francisella*, and *Coxiella*) caused by NCBI blast result was drawn with 1,000 repeated estimated bootstrap values.

For the selected representative reads from *Rickettsia, Anaplasma*, *Francisella*, and *Coxiella,* the phylogenetic tree, as shown in Figure 8, manifests sequences alignment implemented with MEGA 11 software, utilizing the Kimura two-parameter model of Neighbor-Joining method. Table 2 presents the average intraspecies pairwise genetic distances for the four *Rickettsia* and 14 reference sequences retrieved from NCBI, which range from 0.045 to 0.173 and 0.000 to 0.012, respectively. The table also indicates the pairwise divergence (Divergence = 1 − percent identity) between stains, denoted above the diagonal in Table 2.

**Table 2:** Genetic distance (above the diagonal) and pairwise divergence (below the diagonal) in the *Rickettsia* sequences with NCBI reference sequence based on the phylogenetic tree.

	*Rickettsiaceae*.1	*Rickettsiaceae*.2	*Rickettsiaceae*.3	*Rickettsiaceae*.4	MG827281	MF496154	LC602357	MZ292055	EU380782	NR041840	JQ480832	NR025920	LC089861	MF002589	MT509813	OL423537	MT279305	OQ392416
*Rickettsiaceae*.1	—	0.886	0.862	0.847	0.9	0.903	0.903	0.903	0.9	0.9	0.898	0.898	0.893	0.903	0.903	0.903	0.903	0.808
*Rickettsiaceae*.2	0.125	—	0.954	0.879	0.976	0.978	0.978	0.978	0.976	0.976	0.978	0.973	0.968	0.973	0.973	0.973	0.973	0.806
*Rickettsiaceae*.3	0.152	0.045	—	0.859	0.956	0.959	0.959	0.959	0.956	0.956	0.959	0.954	0.956	0.954	0.954	0.954	0.954	0.818
*Rickettsiaceae*.4	0.173	0.135	0.155	—	0.893	0.896	0.896	0.896	0.893	0.893	0.9	0.9	0.891	0.896	0.896	0.896	0.898	0.91
MG827281	0.108	0.025	0.043	0.116	—	0.998	0.998	0.998	0.995	0.995	0.993	0.993	0.988	0.993	0.993	0.993	0.993	0.825
MF496154	0.105	0.022	0.04	0.114	0.002	—	1	1	0.998	0.998	0.995	0.995	0.99	0.995	0.995	0.995	0.995	0.828
LC602357	0.105	0.022	0.04	0.114	0.002	0	—	1	0.998	0.998	0.995	0.995	0.99	0.995	0.995	0.995	0.995	0.828
MZ292055	0.105	0.022	0.04	0.114	0.002	0	0	—	0.998	0.998	0.995	0.995	0.99	0.995	0.995	0.995	0.995	0.828
EU380782	0.108	0.025	0.043	0.116	0.005	0.002	0.002	0.002	—	0.995	0.993	0.993	0.988	0.993	0.993	0.993	0.993	0.828
NR041840	0.108	0.025	0.043	0.116	0.005	0.002	0.002	0.002	0.005	—	0.993	0.993	0.988	0.993	0.993	0.993	0.993	0.83
JQ480832	0.111	0.022	0.04	0.108	0.007	0.005	0.005	0.005	0.007	0.007	—	0.995	0.99	0.995	0.995	0.995	0.995	0.828
NR025920	0.111	0.028	0.045	0.108	0.007	0.005	0.005	0.005	0.007	0.007	0.005	—	0.99	0.995	0.995	0.995	0.995	0.833
LC089861	0.117	0.033	0.043	0.119	0.012	0.01	0.01	0.01	0.012	0.012	0.01	0.01	—	0.99	0.99	0.99	0.99	0.833
MF002589	0.105	0.028	0.045	0.114	0.007	0.005	0.005	0.005	0.007	0.007	0.005	0.005	0.01	—	1	1	0.995	0.83
MT509813	0.105	0.028	0.045	0.114	0.007	0.005	0.005	0.005	0.007	0.007	0.005	0.005	0.01	0	—	1	0.995	0.83
OL423537	0.105	0.028	0.045	0.114	0.007	0.005	0.005	0.005	0.007	0.007	0.005	0.005	0.01	0	0	—	0.995	0.83
MT279305	0.105	0.028	0.045	0.111	0.007	0.005	0.005	0.005	0.007	0.007	0.005	0.005	0.01	0.005	0.005	0.005	—	0.83
OQ392416	0.215	0.219	0.205	0.088	0.193	0.189	0.189	0.189	0.189	0.186	0.189	0.183	0.183	0.186	0.186	0.186	0.186	—

Divergence = 1 − percent identity.

**Figure 9:**
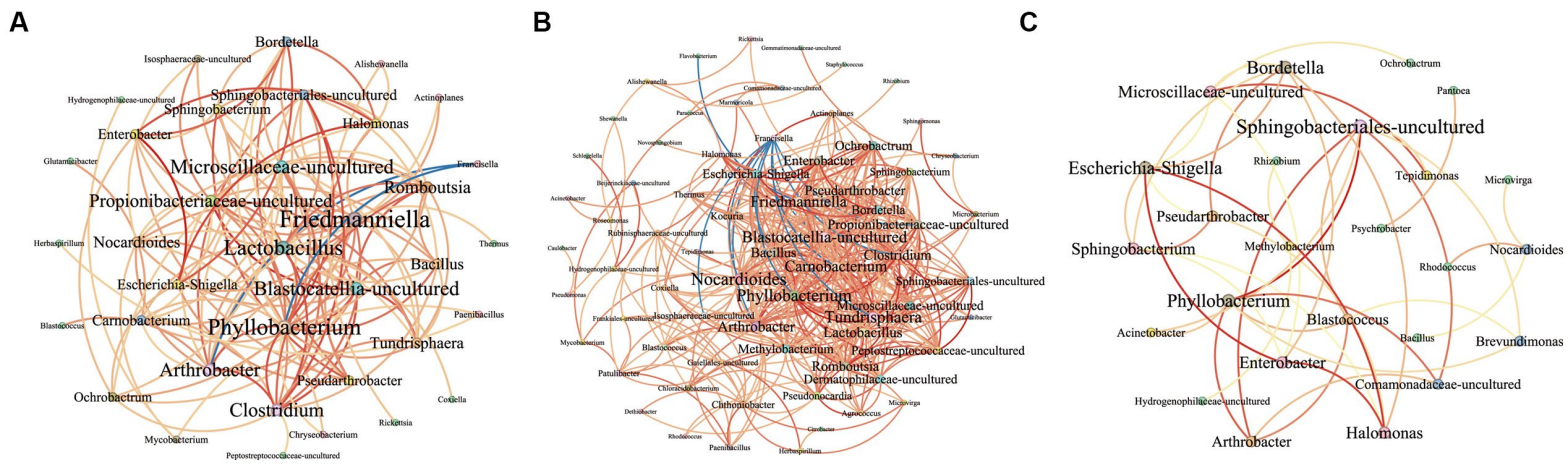
This image presents an interrelationship network among various bacterial genera at the *genus* level. Negative and positive correlations are represented by blue and red lines, respectively. The gradation from yellow to red in the lines signifies the degree of correlation, with a deeper red indicating a stronger correlation. The size of the bacterial names corresponds to the number of relationships each has within the network, with larger names indicating a greater number of connections. **(A)** The network shows the correlations of bacteria in all samples. **(B)** The network shows the correlations of bacteria in Group A. **(C)** The network shows the correlations of bacteria in Group B.

The network, as depicted in Figure 9A, illustrates the correlational relationship among bacterial genera based on co-occurrence patterns, with varying line colors representing different relationships between two genera. The network consists of 36 nodes and 133 links, with only two instances of negative correlation noted between *Francisella* and *Phyllobacterium* (*r* = −0.65), and between *Francisella* and *Arthrobacter* (*r* = −0.63). There are 22 pairs exhibiting strong positive correlation (*r* > 0.8), with the top three pairs being *Sphingobacteriales-uncultured* and *Phyllobacterium* (*r* = 0.88), *Blastocatellia-uncultured* and *Propionibacteriaceae-uncultured* (*r* = 0.92), and *Enterobacter* and *Escherichia-Shigella* (*r* = 0.98). The detailed results for all bacteria are provided in Supplementary Table 5. In order to examine the correlation between bacterial genera across groups, we plotted network diagrams for Group A (Figure 9B) and Group B (Figure 9C). And there are 390 correlational relationships among bacteria in Group A and more than 350 in Group B, which are shown in Supplementary Tables 6, 7.

## 4 Discussion

Ticks, widely distributed blood-sucking parasitic arthropod, can be categorized into feeding ticks and questing ticks based on their living environment. While numerous studies have compared microbial compositions of various tick species morphologically (Krige et al., 2021), the presence of definitive differences in composition and diversity of abundance between feeding and questing ticks remains a debate topic. Our study, however, elucidates the presence of diverse compositions within groups, indicating the value in comparing two ticks of the same species and from the same sampling site.

*Ixodidae* species are widely distributed in Wuwei city, such as *Hyalurus asiaticus*, *Haemaphysalis qinghaiensis*, *Dermacentor nuttalli*, and *Lycoris argenteus* (Sun et al., 2016), and our molecular biological results for *Dermacentor nuttalli* align with the ecological species at the sampling site. In contrast to a study conducted in Inner Mongolia (Jiao et al., 2021), which detected the bacterial genera *Rickettsia*, *Anaplasma*, and *Coxiella* in *D. nuttalli*, our research detected the same bacteria in *D. nuttalli*, indicating that this species of *D. nuttalli* is predisposed to carry these bacteria. However, the detection rate of *Anaplasma* (4.67%, 7/150) in our results was lower than in the former study, where *Anaplasma* spp. was consistently reported and speculated to be a dominant regional species in Inner Mongolia (Jiao et al., 2021). This suggests that greater attention should be directed toward *D. nuttalli* in Wuwei City for comprehensive microorganism investigation, and the sample size, sampling points, and range of ticks should be expanded in further research.

In the relative abundance graph (Figure 3A), the *family Rikettsiaceae* constitutes the largest proportion, accounting for 81.11% in Group A and 87.71% in Group B, primarily including the genera *Rickettsia* and *Ac37b* at the *genus level*. *Rickettsia* peculiarly grows only within the cytoplasm of eucaryotic cells without producing exotoxins, and destructs host-cells from the inside, which are at high risk of infection as obligate intracellular bacteria first described by *Ricketts* in 1909 (Ricketts, 1909). After dengue infection, rickettsial diseases have been reported as the second most common cause of non-malarial febrile infectious illness in Southeast Asia (Acestor et al., 2012). One study reported a significantly higher prevalence of *Rickettsia* spp. in the *genus Dermacentor* than in the other tick species (Del Cerro et al., 2022), and a Malaysian study demonstrated that *Rickettsia RF2125* plays a dominant role in both feeding [31.8% (7/22)] and questing [25.0% (19/76)] ticks (Kho et al., 2019), which aligns with our research that *Rickettsia* exhibited the highest relative abundance and implying a propensity for *Dermacentor* to harbor *Rickettsia* spp.

The blood-feeding behavior of ticks may explain the observed statistical difference between *Friedmanniella* and *Bordetella* in detected bacteria, which may be largely attributable to the host’s herbivorous traits and potential respiratory diseases. Utilizing the Welch’s *t*-test and LEfSe to detect possible *genus*-level differences, we found that *Friedmanniella*, a *genus* belonging to the *Propionibacteriaceae family* frequently detected in plant samples, exhibits significant differences between groups. Group A contained more reads than Group B (*p* = 0.015). Likewise, the *Bordetella*, an animal-associated *genus* known to cause whooping cough (*Bordetella pertussis*), had more reads in Group A and the difference was significant (*p* = 0.030). Extensive research into *Bordetella* has focused on the respiratory pathogens *B. pertussis* and *B. bronchiseptica* (Stevenson and Roberts, 2003), and they cause respiratory illness in children and animals including mice, dogs, pigs, and poultry, respectively (Trainor et al., 2015; Nieves and Heininger, 2016). Transmission of *Bordetellae* is typically attributed to respiratory droplets from coughing patients (Nieves and Heininger, 2016) and does not normally invade beyond the respiratory tract (Stevenson and Roberts, 2003). The bacteria can colonize in the respiratory tract, blood, and body fluids (such as cerebrospinal fluid; Liao et al., 2022), and *B. bronchiseptica* persist in a variety of environments outside the host, including water and on surfaces (Porter et al., 1991). Though no current reports manifest the presence of *Bordetella* in arthropods, our findings imply that *Dermacentor nuttalli* could be a potential vector for *Bordetella*, contradicting other reports that solely suggest transmission and infectious in mammals (Mattoo and Cherry, 2005). However, 75% ethyl alcohol may not be able to kill all pathogens on the surface of ticks, resulting in bacteria contaminating their surfaces, which requires further study to verify ticks as the vector of *Bordetella*.

Although our research detected three ticks for pathogens in one sample, it is possible that pathogens are co-infected in one tick. Tick-borne co-infections result from infection by genetically distinct pathogens (Cutler et al., 2021). In our study, the simultaneous detection rate of *Coxiella*, *Francisella*, and *Rickettsia* was 79.59% (39/49), as demonstrated in Figure 3C, and the prevalence indicates that these genera are remarkably commonplace in multiple tick species and possibly function as symbionts within ticks (Abreu et al., 2019). Indeed, co-infections and symbiote relationships are frequently observed among arthropods such as ticks, ranging from closely related variants within the same species to highly diverse pathogens, including parasites, bacteria, and viruses (Cutler et al., 2021). One research indicates a substantial presence of 90% prevalence rate (153/170) of *R. africae* in *Amblyomma variegatum* ticks and harbored a combination of pathogens, notably including *Coxiella burnetii* among others (Ehounoud et al., 2016). Furthermore, corroborative research has provided evidence of simultaneous infection with two different types of *Rickettsia* (namely *Rickettsia* spp. and *Rickettsiella* spp.) in cases (Raulf et al., 2018). Other bacteria of notable prevalence are *Coxiella* and *Francisella*, which have been reported to exist symbiotically in ticks such as *Rickettsia*-like, *Coxiella*-like, and *Francisella*-like endosymbiont (Song et al., 2022), and potentially play a crucial role in tick development by supplying B vitamins (Brenner et al., 2021).

Networks show the interrelationship between bacteria and Supplementary Tables 6, 7 present the detailed correlation results of groups. The *Francisella* appears to be inhibited or compete with 10 bacterial genera such as *Carnobacterium* (*r* = −0.77) and *Phyllobacterium* (*r* = −0.70), and the *Flavobacterium* inhibit *Friedmanniella* bacteria in Group A (*r* = −0.79). Conversely, no such competitive relations are observed in Group B. This phenomenon may be related to the competition between bacteria inherently present (such as *Francisella*) in questing ticks and those acquired from the host’s blood by parasitic ticks. Notably, strong positive correlation relationships (*r* > 0.8) are evident in both groups with the result between *Phyllobacterium* and *Arthrobacter* (Group A = 0.81, Group B = 0.86), *Phyllobacterium* and *Sphingobacteriales-uncultured* (Group A = 0.84, Group B = 0.99). And the bacteria *Halomonas*, *Escherichia-Shigella,* and *Enterobacter* have strong positive relationships in two groups, which three bacteria may promote growth mutually. It has been also reported that the three bacteria can be detected in animals simultaneously, such as cows, *Haemonchus contortus*, and *Scylla paramamosain* (Zhang et al., 2018; Chen et al., 2020; Mafuna et al., 2021). Interestingly, *Bordetella* has strong positive correlativity with the bacteria *Halomonas* (*r* = 0.81), *Enterobacter* (*r* = 0.90), and *Escherichia-Shigella* (*r* = 0.92) in Group A. Three bacteria, with more abundance in Group A, probably promoted the colonization and growth of *Bordetella*, which may related to the phenomenon that the *Bordetella* has significant differences between two groups.

Our study acknowledges that the evidence focusing on just one sampling site, one city, and the same tick species may be less sufficient. Additionally, the interactive mechanisms of symbiotic bacteria were not examined and verified. Nonetheless, our work yields significant findings. The bacterial genera *Friedmanniella* and *Bordetella* have statistical differences between parasitic and questing ticks, and the interrelationships in bacteria are different in diverse lifestyles ticks. In conclusion, our comparison of parasitic and questing ticks provides unique perspective into diversities in pathogen proportion and microbial communities, leveraging the 16S rRNA gene sequencing and analysis capabilities of NGS. In further research, the sampling range should be expanded and the mechanisms between bacterial genera warrant resolution and optimization.

## 5 Conclusion

The conducted experimental investigations have elucidated differences in the causative agents present in two lifestyle categories of ticks within Wuwei City. It was found that microorganisms demonstrate significant variations between parasitic and questing ticks, with the potential for bacterial genera to either inhibit or promote each other within diverse tick populations. These results underscore significance of lifestyle-based classification in researching potential pathogens.

## Data availability statement

The datasets presented in this study can be found in online repositories. The names of the repository/repositories and accession number(s) can be found below: NCBI—PRJNA1015185.

## Ethics statement

The manuscript presents research on animals that do not require ethical approval for their study.

## Author contributions

LZ: Data curation, Formal analysis, Writing – original draft, Writing – review & editing. JH: Formal analysis, Investigation. QZ: Methodology, Supervision. ZH: Methodology, Writing – review & editing. S-WS: Methodology, Resources, Writing – review & editing. RL: Data curation, Resources, Writing – review & editing. R-SL: Investigation, Visualization, Writing – review & editing. W-KZ: Project administration, Validation, Writing – review & editing. Y-HW: Methodology, Software, Writing – review & editing. L-LX: Investigation, Supervision, Writing – review & editing. Z-HL: Investigation, Software, Writing – review & editing. Z-JS: Conceptualization, Funding acquisition, Supervision, Writing – review & editing.

## Footnotes

^1^http://huttenhower.sph.harvard.edu/galaxy

^2^http://blast.ncbi.nlm.nih.gov/blast.cgi

^3^https://gephi.org
