# Supplementary material for: Differential microbial composition in parasitic vs. questing ticks based on 16S next-generation sequencing
Source: Front Microbiol. 2023 Dec 7;14:1264939. doi: 10.3389/fmicb.2023.1264939 (PMC10773790; doi:10.3389/fmicb.2023.1264939)
Supplement: Supplementary file 1 [file Data_Sheet_1.zip › Supplementary Material.docx]

Supplementary Material

# Supplementary Figures and Tables

## Supplementary Figure


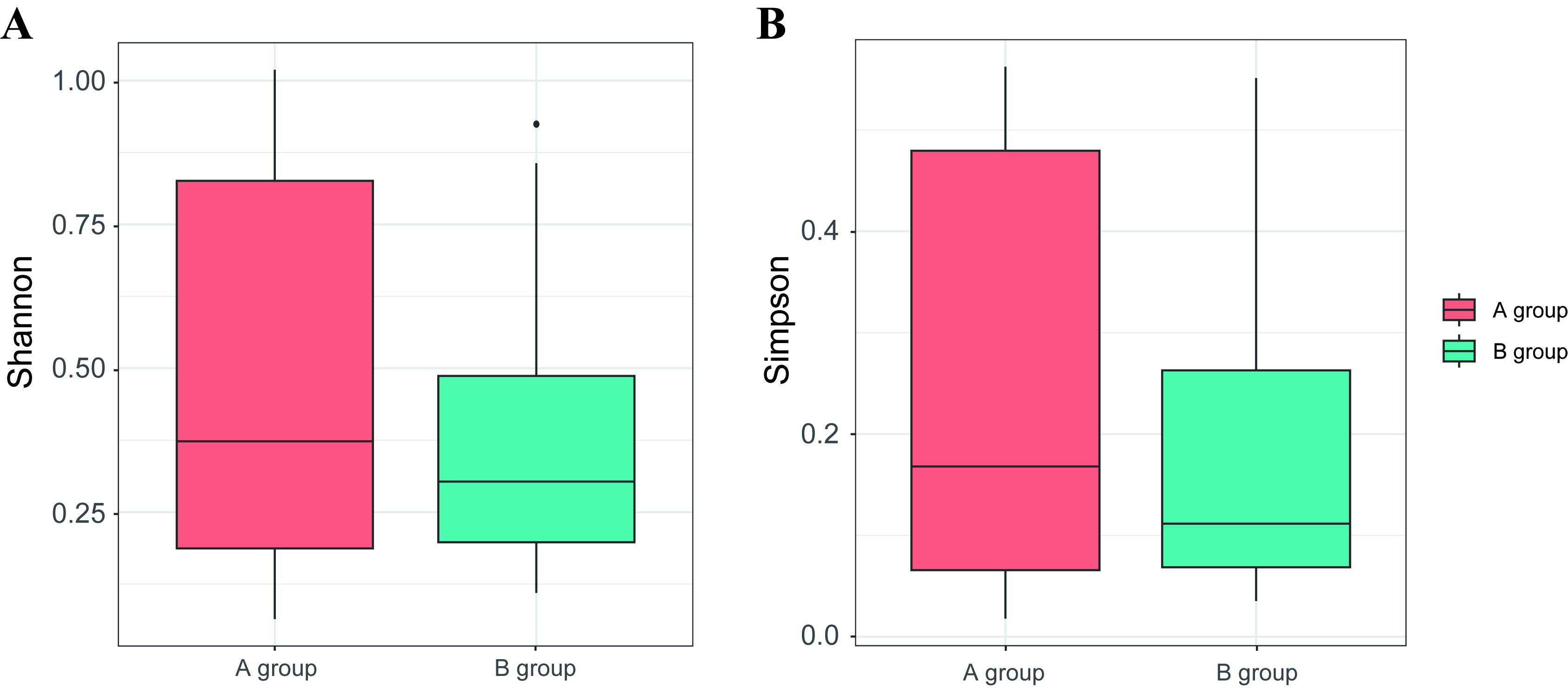


**Supplementary Figure 1** The box plot shows a comparison of the index of Shannon (*p* = 0.375) and Simpson (*p* = 0.441) between groups.

## Supplementary Tables

**Supplementary Table 1** The co-infection rate of five pathogens.

| Prevalence of tick-borne pathogens in individual samples | |
| --- | --- |
| Pathogen | Number of individual ticks positive |
| Single |  |
| Rickettsia | 49 (32.67%) |
| Anaplasma | 7 (4.67%) |
| Lyme spirochete | 0 |
| Bassi worm | 0 |
| Bartonella | 0 |
| Double |  |
| Rickettsia,Anaplasma | 7 (4.67%) |

**Supplementary Table 2** The statistics of sequencing results.

| Bacterial 16S NGS statistics | | | |
| --- | --- | --- | --- |
| Sequence statistic | Raw (unprocessed)reads | Sequence Length | Processed 16S sequences |
| Average | 162,731 | 395 | 109,607 |
| Minimum | 74,332 | 250 | 42,877 |
| Maximum | 417,497 | 468 | 257,450 |
| Total | 8,136,535 | 1230 | 5,480,349 |
| No. of ZOTUs |  |  | 3,070 |

**Supplementary Table** **3** The Wilcoxon rank sum test of the Alpha diversity index between groups at the *family* level.

| Wilcoxon rank sum test | | | | | | | | |
| --- | --- | --- | --- | --- | --- | --- | --- | --- |
| Alpha diversity | Shannon | Simpson | Pielou | goods_covers | observed_species | Richness | Chao1 | Ace |
| *W* | 359 | 353 | 349 | 288 | 386 | 386 | 382.5 | 374 |
| *p*-value | 0.375 | 0.441 | 0.488 | 0.639 | 0.156 | 0.156 | 0.177 | 0.237 |

**Supplementary Table 4** The Alpha diversity index between groups after removing *Rickettsiaceae* at the *family* level.

| The Alpha diversity index between groups | | | | | | | | |
| --- | --- | --- | --- | --- | --- | --- | --- | --- |
|  | Shannon | Simpson | Pielou | goods_covers | observed_species | Richness | Chao1 | Ace |
| Group A | 1.044 | 0.520 | 0.203 | 0.99 | 172 | 172 | 182.688 | 180.976 |
| Group B | 1.363 | 0.628 | 0.267 | 0.99 | 164 | 164 | 164 | 164.265 |

# Supplementary Nucleotide Sequences

## COI nucleotide sequence

>COI gene sample

GTAATATATGATGAGCCCATACAATAAATCCGAGTAAACCAATTGCTGCTATTGCATAAATTATTCCTAAGTTTCCAAAAGGTTCCTTTTTACCTGTTCTAAAGCAAATAATTTGTGAAATTATTCCAAATCCTGGAAGAATTAAAATGTAGACCTCGGGGTGCCCAAAAAATCAAAATAGATGTTGATATAAAATTGGATCTCCCCCCCCTGAAGGGTCAAAGAATGATGTATTAAAATTTCGATCTGTTAATAATATGGTAATTGCYCCTGCTAAAACAGGTAAGGAAAGTAAAAGTAAAATTGCAGTAATTAATACTGATCAAACAAATAAAGGTATACGTTCAAGAGTTATTCCAATTGATCGTATGTTAATAATTGTAGTAATGAAATTAATTGCTCCTAGAATTGATGATGCCCCTGCTAAATGAAGAGAGAAAATTGCTAAATCTACTGAAGGGCCATAATGTGATAAATTGGAAGATAAAGGAGGGTAAACTGTTCAACCAGTCCCTGCTCCTGATTCAATTAAAGAAGAATTGATTAATAAGAATAGTGAAGGTGGAAGTAACCAGAATCTTATATTATTTATTCGGGGAAAAGCTATATCTGGAGCACCTAATATAATTGGGACAAGCCAATTCCCGAACCCCCCGATTATAATAGGTATAACTATAAAAAAAATTATAATGAAAGCGTGAGCAGTTACAATTACGTTATAAATTTGATCATTTCCAATTAATGTCCCAGGTTGRCTAAGTTCTATTCGAATAAGAATTCTTATTCTTATTCCTATTATTCCAGCTCAGCTTCCAAAA

## *Rickettsia* nucleotide sequences

### >*Rickettsia* 1 sequence

GTGCCAGCAGCCGCGGTAAGACGGAGGGGGCTAGCGTTGTTCGGAATTACTGGGCGTAAAGAGTGCGTAGGCGGTTTAGTAAGTTGGAAGTGAAAGCCCGGGGCTTAACCTCGGAATTGCTTTCAAAACTACTAATCTAGAGTGTAGTAGGGGATGATGGAATTCCTAGTGTAGAGGTGAAATTCTTAGATATTCGGAGGAACACCAGTGGCGAAGGCGGCTCACTGGACCATTACTGACGCTCAGGTGCGAAAGCGTGGGGAGCAAACAGGATTAGATACCCTGGTAGTCCACGCCGTAAACGATGTCGACTTGGAGGTTGTGCCCTTGAGGCGTGGCTTCCGGAGCTAACGCGTTAAGTCGACCGCCTGGGGAGTACGGCCGCAAGGTTAAAACTCAAAGGAATTGACGG

### >*Rickettsia* 2 sequence

GTGCCAGCCGCCGCGGTAAGACGGAGGGGGCTAGCGTTGTTCGGAATTACTGGGCGTAAAGAGTGCGTAGGCGGTTTAGTAAGTTGGAAGTGAAAGCCCGGGGCTTAACCTCGGAATTGCTTTCAAAACTACTAATCTAGAGTGTAGTAGGGGATGATGGAATTCCTAGTGTAGAGGTGAAATTCTTAGATATTAGGAGGAACACCGGTGGCGAAGGCGATCATCTGGGCTACAACTGACGCTGATGCACGAAAGCGTGGGGAGCAAACAGGATTAGATACCCTGGTAGTCCACGCCGTAAACGATGAGTGCTAGATATCGGAAGATTCTCTTTCGGTTTCGCAGCTAACGCATTAAGCACTCCGCCTGGGGAGTACGGTCGCAAGATTAAAACTTAAATGAATTGACGG

### >*Rickettsia* 3 sequence

GTGCCAGCAGCCGCGGTAATACAGAGGGTGCAAGCGTTGTTCGGAATTATTGGGCGTAAAGGGCGCGTAGGCGGCCTGACAAGTCGAATGTGAAATCCCAGGGCTCAACCCTGGAAGTGCATCCGAAACTACTAATCTAGAGTGTAGTAGGGGATGATGGAATTCCTAGTGTAGAGGTGAAATTCTTAGATATTAGGAGGAACACCGGTGGCGAAGGCGATCATCTGGGCTACAACTGACGCTGATGCACGAAAGCGTGGGGAGCAAACAGGATTAGATACCCTGGTAGTCCACGCCGTAAACGATGAGTGCTAGATATCGGAAGATTCTCTTTCGGTTTCGCAGCTAACGCATTAAGCACTCCGCCTGGGGAGTACGGTCGCAAGATTAAAACTTAAAGGAATTGACGG

### >*Rickettsia* 4 sequence

GTGCCAGCAGCCGCGGTAAGACGGAGGGGGCTAGCGTTGTTCGGAATTACTGGGCGTAAAGAGTGCGTAGGCGGTTTAGTAAGTTGGAAGTGAAAGCCCGGGGCTCAACCCCGAGTCTGCAGTGGGTACGGGCAGACTAGAGTGCAGTAGGGGAGACTGGAATTCCTGGTGTAGCGGTGAAATGCGCAGATATCAGGAGGAACACCGATGGCGAAGGCAGGTCTCTGGGCTGTTACTGACGCTGAGGAGCGAAAGCATGGGGAGCGAACAGGATTAGATACCCTGGTAGTCCACGCCGTAAACGATGAGTGCTAGATATCGGAAGATTCTCTTTCGGTTTCGCAGCTAACGCATTAAGCACTCCGCCTGGGGAGTACGGTCGCAAGATTAAAACTCAAATGAATTGACGG

## *Anaplasma* nucleotide sequence

>*Anaplasma* sequence

GTGCCAGCAGCCGCGGTAATACGGAGGGGGCAAGCGTTGTTCGGAATTATTGGGCGTAAAGGGCATGTAGGCGGTTTGGTAAGTTAAAGGTGAAATACCAGGGCTTAACCCTGGGGCTGCTTTTAATACTGCAGGACTAGAGTCCGGAAGAGGATAGCGGAATTCCTAGTGTAGAGGTGAAATTCGTAGATATTAGGAGGAACACCAGTGGCGAAGGCGGCTGTCTGGTCCGGTACTGACGCTGAGGTGCGAAAGCGTGGGGAGCAAACAGGATTAGATACCCTGGTAGTCCACGCTGTAAACGATGAGTGCTGAATGTGGGGGCTTTTGCCTCTGTGTTGTAGCTAACGCGTTAAGCACTCCGCCTGGGGACTACGGTCGCAAGACTAAAACTCAAATGAATTGACGG

## *Francisella* nucleotide sequence

>*Francisella* sequence

GTGCCAGCCGCCGCGGTAATACGGGGGGTGCAAGCGTTAATCGGAATTACTGGGCGTAAAGGGTCTGTAGGTGGTTTGTTAAGTCAGATGTGAAAGCCCAGGGCTCAACCTTGGAACTGCATTTGATACTGGCAAACTAGAGTACGGTAGAGGAATGGGGAATTTCTGGTGTAGCGGTGAAATGCGTAGAGATCAGAAGGAACACCAATGGCGAAGGCAACATTCTGGACCGATACTGACACTGAGGGACGAAAGCGTGGGGATCAAACAGGATTAGATACCCTGGTAGTCCACGCTGTAAACGATGAGTACTAGCTGTTGGAGTCGGTGTAAAGGCTCTAGTGGCGCAGCTAACGCGATAAGTACTCCGCCTGGGGACTACGGCCGCAAGGCTAAAACTTAAATGAATTGACGG

## *Coxiella* nucleotide sequences

### >*Coxiella* 1 sequence

GTGCCAGCAGCCGCGGTAATACAGAGAGTGCAAGCGTTAATCGGAATCACTGGGCGTAAAGTGCGCGTAGGTGGATGTTTAAGTCGGATGTGAAAGCCCTGGGCTTAACCTAGGAACTGCATCCGATACTGGATGTCTCGAGTATGGTAGAGGGAAGTGGAATTTCCGGTGTAGCGGTGAAATGCATAGATATCGGAAAGAACACCAGTGGCGAAGGCGGCTTCCTGGACTAATACTGACACTAAGGCGCGAAAGCGTGGGGAGCAAACAGGATTAGATACCCTGGTAGTCCACGCCCTAAACGATGTCAACTAGCTGTTGGGGCCTTCGGGCCTTAGTAGCGCAGCTAACGCGTGAAGTTGACCGCCTGGGGAGTACGGTCGCAAGATTAAAACTTAAATGAATTGACGG

### >*Coxiella* 2 sequence

GTGCCAGCAGCCGCGGTAATACGGAGGGGGCTAGCGTTGTTCGGAATTACTGGATGTCTAGAGTCTTGTAGAGGGAAGTGGAATTTCCGGTGTAGCGGTGAAATGCATAGATATCGGAAAGAACACCAGTGGCGAAGGCGGCTTCCTGGACTAATACTGACACTAAGGCGCGAAAGCGTGGGGAGCAAACAGGATTAGAGACCCTGGTAGTCCACGCCGTCAACGATGAGAACTAGCTGTTGGGGAGTTTACTTTCTAGTAGCGAAGCTAACGCGTTAAGTTCTCCGCCTGGGGAGTACGGCCGCAAGGTTAAAACTCAAAGGAATTGACGG

### >*Coxiella* 3 sequence

GTGCCAGCCGCCGCGGTAATACAGAGAGTGCAAGCGTTAATCGGAATCACTGGGCGTAAAGTGCGCGTAGGTGGATGTTTAAGTCGGATGTGAAAGCCCTGGGCTTAACCTAGGAACTGCATCCGATACTGGATGTCTCGAGTATGGTAGAGGGAAGTGGAATTTCCGGTGTAGCGGTGAAATGCATAGATATCGGAAAGAACACCAGTGGCGAAGGCGGCTTCCTGGACTAATACTGACACTAAGGCGCGAAAGCGTGGGGAGCAAACAGGATTAGAGACCCTGGTAGTCCACGCCGTCAACGATGAGAACTAGCTGTTGGGGAGTACGGTCGCAAGATTAAAACTCAAAGGAATTGACGG
